# Retrospective and Prospective

**Published:** 1892-01

**Authors:** 


### Editorial.

### RETROSPECTIVE AND PROSPECTIVE.

THIS number commences a new year and the thirteenth volume of this journal.

It becomes, therefore, an appropriate moment to restate the purpose of its existence and to review what has been done in the past. It cannot be out of place here to define the sentiment that every profession should have its periodical literature under its own control. This is necessary, not only for the freest development of thought, but for the growth of high patriotic spirit. The latter is probably more important than the former, for without the culture of the higher elements of professional life the whole aspect of professional effort must become dwarfed. The recognition of these principles, and the knowledge of the fact that dentistry for years had not, in this country, possessed under its own control a journal devoted to its interests, led to the establishment of the *Independent Practitioner*, of which this journal is the successor.

To this sentiment of loyalty, and to the necessity for the existence of independent journalism, we are uncompromisingly bound, and hold that no other class is really entitled to the free support of our profession.

We have the satisfaction of thankfully acknowledging the endorsement by the many leading men of every section who have steadfastly taken this journal. In the trials which attend the beginning of every such enterprise we have had the solace that many of the most intelligent and better men have been our readers, but we want more than this. We ask that every one should come to us, not only for the reason that the greater the number of our patrons the more liberal can we conduct our affairs, but that wholesome pride requires that each and all should be found among our readers.

In the past we have held to our colors, and have in a painstaking and faithful manner carried on the work of publishing the matter placed under our control. We also have the gratification of knowing that this journal has given satisfaction to those who have been its regular readers, and this sentiment has been deepened by the knowledge that the subscribers are so largely composed of the most influential men of all parts of this country and of Europe.

An examination of our pages will show that they have contained a very large quantity of the most useful matter and that many of the papers have become of great value, which is attested by the frequency with which they have been copied. The discussions of the papers, to those who have carefully read them, have furnished a large amount of that living kind of information which comes spontaneously from men who are in touch with dental work.

The present period is, we believe, from various indications, the commencement of a new era in the experience of this journal. The circulation has been steadily increasing, and there are notable instances of renewed interest in its work.

In view of this increasing interest and of the additional strength of which it gives evidence, the promoters of this journal feel justified in making an earnest appeal to their fellows to join hands with them in the effort to enlarge the usefulness of the trust resting upon them.

